# Neural reflex control of vascular inflammation

**DOI:** 10.1186/s42234-020-0038-7

**Published:** 2020-01-31

**Authors:** A. S. Caravaca, M. Centa, A. L. Gallina, L. Tarnawski, P. S. Olofsson

**Affiliations:** 1 Laboratory of Immunobiology, Center for Bioelectronic Medicine, Department of Medicine, Solna, Karolinska Institutet, Stockholm, Sweden; 2 Center for Biomedical Science and Bioelectronic Medicine, The Feinstein Institute for Medical Research, Manhasset, NY 11030 USA

## Abstract

Atherosclerosis is a multifactorial chronic inflammatory disease that underlies myocardial infarction and stroke. Efficacious treatment for hyperlipidemia and hypertension has significantly reduced morbidity and mortality in cardiovascular disease. However, atherosclerosis still confers a considerable risk of adverse cardiovascular events. In the current mechanistic understanding of the pathogenesis of atherosclerosis, inflammation is pivotal both in disease development and progression. Recent clinical data provided support for this notion and treatment targeting inflammation is currently being explored. Interestingly, neural reflexes regulate cytokine production and inflammation. Hence, new technology utilizing implantable devices to deliver electrical impulses to activate neural circuits are currently being investigated in treatment of inflammation. Hopefully, it may become possible to target vascular inflammation in cardiovascular disease using bioelectronic medicine. In this review, we discuss neural control of inflammation and the potential implications of new therapeutic strategies to treat cardiovascular disease.

## Introduction

The immune system responds to microbes and tissue injury and strives to maintain homeostasis by eradicating threats of infection and promoting tissue repair. Invasion and injury are sensed by several mechanisms. A range of immune cells and sensory neurons express receptors for pathogen associated molecular patterns (PAMP), damage associated molecular patterns (DAMP), cytokines, chemokines, irritants, and other infection- and inflammation-associated molecules (Chiu et al. 2012; Rivera et al. 2016). Accordingly, both immune cells and neurons respond to infection and injury to coordinate the inflammatory response and defense from pathogens (Andersson and Tracey 2012; Goehler et al. 2000; Chiu et al. 2013; Baral et al. 2018; Pinho-Ribeiro et al. 2016; Blake et al. 2018). The vasculature plays an important role in anti-microbial defense and tissue healing (Kozarov 2012). Vascular inflammation is also a key factor in the development of atherosclerosis, and blocking pro-inflammatory cytokines may reduce aspects of cardiovascular disease (Hansson and Libby 2006; Ridker et al. 2017a). The interplay between the nervous and immune systems in the pathogenesis of cardiovascular disease is not well understood.

### Inflammation in atherosclerosis

**Fig. 1:**
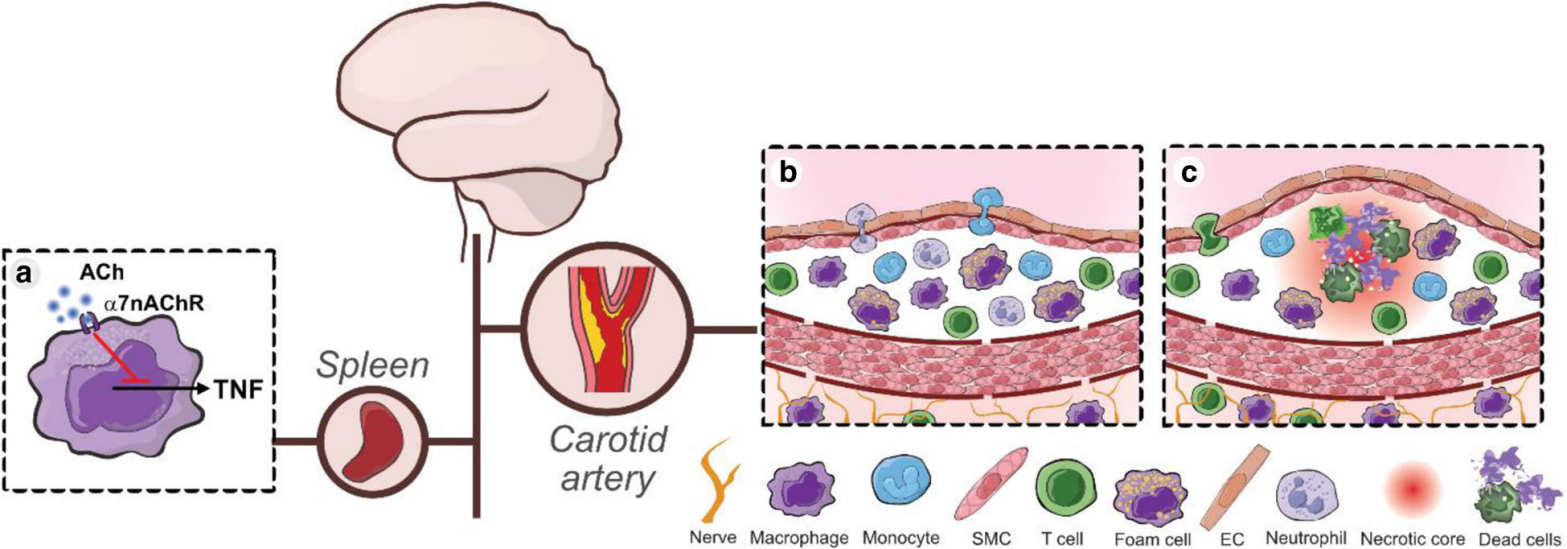
Neural control of vascular inflammation. Neural circuits regulate inflammation and cytokine production. **a** In the inflammatory reflex, acetylcholine (ACh) acts through the alpha 7 nicotinic acetylcholine receptor subunit (α7nAChR) on macrophages to suppress pro-inflammatory cytokines such as TNF. Suggested neuro-immune cross talk in atherosclerosis: **b** The adventitia is innervated and contains immune cells that may interact with other layers of the vascular wall. In the early stages of atherosclerosis, local recruitment of inflammatory cells in the intimal layer of arteries progresses slowly. **c** As atherosclerosis progresses, the inflamed plaque eventually develops a necrotic core which increases plaque vulnerability and the risk of rupture

Atherosclerosis is a major underlying cause of cardiovascular disease, the main cause of death worldwide (Herrington et al. 2016). It is defined by the formation and growth of atheromatous plaques in the arterial walls of medium- and large-size arteries characterized by local lipid accumulation, cell death, and fibrosis (Hansson and Libby 2006). Initially, lipid-laden macrophages accumulate beneath the endothelium and form fatty streaks. This early disease stage is asymptomatic, and progresses slowly with local buildup of inflammatory cells and smooth muscle cells in the intimal layer of arteries. This low-grade inflammation eventually develops into an exocentric thickening in the arterial wall into an atheromatous plaque. The plaque commonly contains a lipid-rich necrotic core, immune cells and cellular debris. It is surrounded by a fibrous cap formed primarily by smooth muscle cells and collagen. Plaques prone to rupture are considered “vulnerable” (Finn et al. 2010). As the disease progresses, local inflammation in the lesion produces radicals, proteases and pro-inflammatory mediators, which may reduce the local integrity of the fibrous cap and increase the risk of plaque rupture, atherothrombosis, and clinical symptoms (Hansson 2005; Tabas 2010; Kojima et al. 2017; Kojima et al. 2019) (Fig. 1).

Vulnerable plaque disruption has also been linked to sheer stress. Non-laminar flow and disturbed shear stress can result in pro-inflammatory gene expression in the vascular wall. (Cunningham and Gotlieb 2005; Chiu and Chien 2011; Cybulsky and Marsden 2014). Areas of the vascular tree that are constantly exposed to turbulent blood flow, such as arterial branching sites, are more susceptible to atherosclerotic plaque formation. Low shear stress promotes endothelial expression of adhesion molecules and recruitment of monocytes (Seneviratne et al. 2013). Together, unfavorable bio-mechanical forces, lipid accumulation, and inflammatory cell infiltration promote plaque formation and development of plaque vulnerability.

Clearly, the molecular mechanisms that underlie development and progression of atherosclerotic plaques are complex (Stemme et al. 1995; Hermansson et al. 2010; Davignon and Ganz 2004). In the following sections we discuss potential strategies to target unresolved inflammation in atherosclerosis, including potential applications of bioelectronic medicine in cardiovascular disease.

### Current treatment strategies for inflammation in atherosclerosis

There has been some success in recent decades in treatment of atherosclerotic cardiovascular disease, with many patients being helped by lipid-lowering therapy and anti-thrombotic drugs (Shapiro and Fazio 2016; Nissen et al. 2005). However, disease events within the treated populations continue to occur, perhaps because some patients are still unable to reach desirable blood levels of low-density lipoprotein cholesterol drugs (Libby 2005; Shapiro et al. 2016; Fava and Montagnana 2018; Ikonomidis and Andreotti 1999).

An emerging potential treatment strategy is targeting inflammation. The recent Canakinumab Anti-Inflammatory Thrombosis Outcomes Study (CANTOS), a randomized trial of blocking interleukin-1β (IL-1β) in cardiovascular disease using canakinumab, showed a significant risk-reduction for adverse cardiovascular events in treated at-risk patients (Ridker et al. 2017b). This trial translates the evidence from experimental studies on the pathogenetic role of inflammation in atherosclerosis (Gisterå and Hansson 2017; Söderström et al. 2018) to the clinic and supports the notion that reducing arterial inflammation in human atherosclerotic cardiovascular disease is beneficial for outcome. Interestingly, in experimental radiation-induced arterial damage, treatment with anti-IL-1β antibodies, i.e. anakinra, significantly reduced inflammation (Christersdottir et al. 2019). These findings suggest that anti-cytokine therapy, e.g. inhibition of IL-1β, may be useful for prevention and treatment of vascular inflammation and atherosclerosis. However, anti-cytokine drugs are currently expensive and commonly require administration by injection (Goehler et al. 2000).

### Neural control of inflammation in atherosclerosis

Research over more than two decades has shown that inflammation is regulated by neural reflexes (Tracey 2002; Olofsson et al. 2012; Pavlov et al. 2018). Infection, inflammation, and specific cytokines elicit signals in sensory nerves (Chiu et al. 2013; Niijima 1996; Watkins et al. 1995; Caravaca et al. 2017a; Zanos et al. 2018; Pinho-Ribeiro et al. 2018), and cytokine release, immune cell activity, antibody production, and lymph flow are regulated by efferent neural signals (Borovikova et al. 2000; Rosas-Ballina et al. 2011; Mina-Osorio et al. 2012; Hanes et al. 2016; Tynan et al. 2019). These processes subject to neural control are important in the pathogenesis of atherosclerosis, and consequently present opportunities for further exploration of potential clinical targets (Gisterå and Hansson 2017; Swirski et al. 2009; Hermansson et al. 2011; Centa et al. 2019) (Fig. 1). The adventitia is innervated and contains immune cells that can interact with other layers of the vascular wall (Stenmark et al. 2013). This anatomical organization suggest that nerves, resident immune cells, and other components involved in vascular inflammation and atherosclerosis may interact. It remains to be investigated whether this potential interaction regulates vascular inflammation and atherosclerosis development.

The *inflammatory reflex* is a neural circuit that regulates inflammation. It has been extensively studied and mechanistically mapped to some degree (Pavlov et al. 2019; Tarnawski et al. 2018). Major components of this neuro-immune circuit include the vagus nerve, the splenic nerve, choline acetyltransferase expressing T-cells (T_ChAT_), and the α7 nicotinic acetylcholine receptor subunit (α7nAChR) (Rosas-Ballina et al. 2011; Olofsson et al. 2016; Cox et al. 2019; Guzik et al. 2007; Wang et al. 2002; Huston et al. 2006; Rosas-Ballina et al. 2008). α7nAChR is expressed by many immune cells, including macrophages, and agonists for the α7nAChR attenuate biosynthesis and release of several pro-inflammatory cytokines, including TNF (Wang et al. 2002).

Interestingly, the α7nAChR is reportedly expressed in human atherosclerotic lesions, and ablation of hematopoietic α7nAChR in mice increased aortic atherosclerosis (Johansson et al. 2014). In addition, α7nAChR^−/−^ atherosclerosis-prone ApoE^−/−^ mice had increased serum CRP and IL-6, as well as increased macrophage cholesterol mass from mouse peritoneal macrophages, compared to α7nAChR^−/−^ x ApoE^wt/wt^ mice (Wilund et al. 2009). Furthermore, administration of GTS-21, a selective α7nAChR agonist, reduced atherosclerotic lesions and plaque size as observed from the aorta of ApoE^−/−^ mice (Al-Sharea et al. 2017). These observations suggest that cholinergic signals to α7nAChR in atherosclerosis can attenuate plaque inflammation and atherosclerosis progression. This is in line with the notion that activation of α7nAChR reduces inflammation in a wide range of animal models of different inflammatory diseases (Steinberg et al. 2016a).

The prototypical agonist for α7nAChR is acetylcholine (ACh). ACh is also the key neurotransmitter of the vagus nerve and biosynthesized by ChAT. Importantly, ACh can also be released to the extracellular space by T_ChAT_, which relay neural signals to α7nAChR-expressing macrophages in spleen and attenuates production of pro-inflammatory cytokines (Olofsson et al. 2012; Rosas-Ballina et al. 2008). Of note, administration of galantamine, an acetylcholinesterase inhibitor, reduced levels of pro-inflammatory cytokines and improved insulin sensitivity in a cohort of patients suffering from the metabolic syndrome (Consolim-Colombo et al. 2017).

Observations in experimental studies indicate that activation of the inflammatory reflex has the potential to regulate initiation of vascular inflammation. For example, cholinergic agonists and vagus nerve stimulation blocked endothelial cell activation, leukocyte extravasation, and recruitment of immune cells to sites of inflammation in a carrageenan air pouch mouse model (Saeed et al. 2005). Exposure to acetylcholine also reduced adhesion molecule expression on TNF-stimulated endothelial cells (Reardon et al. 2013), which supports the observation that cholinergic stimulation of the endothelium limits its activation in inflammation (Saeed et al. 2005).

Interestingly, T_ChAT_ under neural control in spleen release acetylcholine to regulate release of pro-inflammatory cytokines in experimental inflammation (Rosas-Ballina et al. 2011), have the capacity to reduce blood pressure (Olofsson et al. 2016) and control microvascular contraction and T cell extravasation in infection (Cox et al. 2019). These observations further support that components of the inflammatory reflex may participate in the regulation of vascular inflammation. In this way, it is possible that there is neural control of recruitment and activity of immune cells in atherogenesis (Olofsson et al. 2016; Rosas-Ballina et al. 2011). Of particular interest is that pharmacological regulation of vascular contraction can mimic the effects of presence or absence of T_ChAT,_ both in terms of dilatation of tissue arterial trees, extravasation of antigen-specific T cells, and anti-viral activity. In other words, changes in vascular contractility are significant for the efficiency of the anti-microbial defense, extravasation of T cells, and perhaps also for vascular inflammation in atherosclerosis. It will be interesting to investigate whether neural control of T_ChAT_ is important for regulation of vascular inflammation.

Resolution of inflammation is vital in pathogenesis and progression of atherosclerosis (Bäck et al. 2019). A number of recent reports indicate that inflammation resolution is under neural control. Mouse and human vagus nerves produce specialized pro-resolving mediators (SPMs), important effectors in resolution of inflammation (Serhan et al. 2018). Vagotomy in peritonitis-induced mice delayed resolution of inflammation and decreased SPMs (Mirakaj et al. 2014). Electrical vagus nerve stimulation in experimental peritonitis decreased resolution time (Caravaca et al. 2017b). While reports on inflammatory reflex activity and resolution of inflammation in cardiovascular disease are lacking, it was observed that restoration of SPMs in murine atherosclerosis suppresses plaque progression and promotes increased fibrous cap thickness (Fredman et al. 2016).

In the adventitia of medium- and large-size arteries, artery tertiary lymphoid organs (ATLOs), develop in proximity of inflammatory foci (Gräbner et al. 2009). Tertiary lymphoid organs (TLOs) are not formed at fixed locations but instead develop in various inflamed non-lymphoid tissues, often as a result of a non-resolving inflammation (Jones et al. 2016). TLOs regulate local immune responses in chronic inflammation, particularly antibody-mediated responses in health and disease (Jones et al. 2016). For instance, the formation of TLOs in autoimmune disease is suggested to be both beneficial, sequestering cells and limiting their spread throughout the body, and pathogenic, where TLOs could be the source of autoreactive and pro-inflammatory lymphocytes (Shipman et al. 2017). On the other hand, cancer associated TLOs have been linked to a favorable outcome in patients, due to their role of initiating and maintaining an immune response against the tumor (Hiraoka et al. 2016). Intriguingly, some TLO development requires vagus nerve innervation which encourages speculation that neural signals may play a role in development also of ATLOs, and raises the question whether blood vessel innervation regulates vascular immune responses and atherosclerosis development (Olivier et al. 2016). The currently available data warrants investigation of the interplay between perivascular lymphoid structures and vascular innervation in development of atherosclerosis.

Taken together, these observations identify neural control of inflammation and resolution in atherosclerosis as an interesting area of study.

### Vagus nerve stimulation and treatment of experimental cardiovascular disease

Aspects of vagus nerve activity can be assessed by analyzing heart rate variability (HRV) (Villareal et al. 2002). HRV is measured by the variation in the time interval between heartbeats and is an indication of cardiac vagal activity. It has been reported that monitoring of HRV in at-risk cardiovascular patients provides prognostic information beyond traditional risk factor analysis, although data is not unequivocal (Villareal et al. 2002; Tsuji et al. 1994; Lanza et al. 1998). Decreased vagal activity may increase cardiovascular risk in both pre-clinical and clinical experiments (Zhao et al. 2013) supporting a potential role of vagus nerve signaling in precipitation of cardiovascular disease.

Pharmacological and electrical vagus nerve stimulation is currently being investigated in experimental cardiovascular disease (Wang et al. 2019). Acetylcholine and acetylcholine-receptor agonists could potentially be of interest to explore further in the pathobiology of cardiovascular disease since muscarinic acetylcholine receptors (mAChRs) play a role in regulating heart rate, smooth muscle contraction, and in fundamental functions of the central nervous system (Kruse et al. 2014). Furthermore, anti-inflammatory and cardioprotective effects of acetylcholine have been demonstrated in ischemia-reperfusion (I/R)-induced oxidative stress models, in which increased acetylcholine levels have been shown to decrease reactive oxygen species formation when rat cardiomyocytes were exposed to hypoxia/reoxygenation to mimic I/R injury (Miao et al. 2013). Cholinergic drugs (e.g. choline, acetylcholine, pyridostigmine) are being studied in treatment of cardiovascular disease (Liu et al. 2019). For example, choline protected against ischaemia-induced arrhythmias (Wang et al. 2012a) and prevented cardiac hypertrophy induced by angiotensin II (Wang et al. 2012b). Acetylcholine is already being investigated as a novel target in drug development for a number of diseases (e.g. Alzheimer’s disease, schizophrenia, type 2 diabetes) (Kruse et al. 2014). Pyridostigmine is a cholinesterase inhibitor, which has been shown in recent studies to restore baroreflex sensitivity, improve heart rate variability, and improve peripheral vascular endothelial function in rats with myocardial infarction (Gavioli et al. 2014; de La Fuente et al. 2013; Liu et al. 2015; Qin et al. 2014). While the use of cholinergic drugs is currently limited in the clinic, pre-clinical studies indicate modulation of vagal nerve activation could have potentially cardiovascular protective effects. Vagus nerve stimulation may be an alternative to pharmacological treatment for activation of α7nAChR or other components of the inflammatory reflex (Eberhardson et al. 2019a; Eberhardson et al. 2019b). VNS ameliorated inflammation in a number of experimental disease models such as endotoxemia, rheumatoid arthritis, inflammatory bowel disease, and kidney ischemia-reperfusion injury (Borovikova et al. 2000; Steinberg et al. 2016a; Levine et al. 2014; Bonaz et al. 2017). Vagus nerve stimulation reduced myocardial infarct size by a mechanism that requires the α7nAChR in rats (Kiss et al. 2017), and decreased inflammation in myocardial ischemia-reperfusion injury under experimental conditions (Brack et al. 2013; Premchand et al. 2014; Wang et al. 2012c; Bernik et al. 2002). There is encouraging data from a few small studies that used implanted vagus nerve stimulators to treat rheumatoid arthritis (Koopman et al. 2016) and Crohn’s disease (Eberhardson et al. 2019a; Bonaz et al. 2016), demonstrating proof-of-concept that this technology may be feasible in clinical use, although additional data is required to sufficiently establish beneficial clinical effects in people with inflammatory diseases.

Attenuation of excessive inflammation can reduce the risk of cardiovascular disease as evidenced by a plethora of animal studies and the CANTOS trial (Libby et al. 2019). Because electrical activation of the inflammatory reflex can reduce pro-inflammatory cytokine levels, it would be interesting to investigate VNS in experimental atherosclerosis. Progression of atherosclerosis is slow and long-term experiments with a duration of many weeks or months are required to properly study effects and mechanisms. It has been challenging to study the effects of VNS in the well-established animal models of atherosclerosis because it requires implantation of VNS electrodes that maintain integrity and functionality of the relatively small interface between the cervical vagus nerve and the electrode over extended periods of time. Chronic interfacing with the peripheral nerves such as the vagus nerve is being explored (Caravaca et al. 2017a), however, implementation in mouse experimental disease models has been difficult. The use of mice is important because the models are well-characterized and permit efficient mapping of mechanism using genetic tools. Regrettably, suitable technology to perform peripheral nerve stimulation in mice over long periods of time in a reasonably practical way is, to the best of our knowledge, not yet available.

### Future perspectives of experimental bioelectronic medicine

The concept of neural reflex regulation of inflammation postulates that the body senses inflammatory processes through a sensory arc, and uses this information to modulate efferent signals through the motor arc. Thus, it may be possible to develop devices and algorithms to monitor cytokine levels and discriminate between different types of inflammation continuously and in real time. Such devices may enable new approaches in inflammation research and provide important new insights into the dynamics of inflammation in health and disease.

Recording of electrical vagus nerve activity has revealed that intraperitoneal injection of pro-inflammatory cytokines TNF and IL-1, respectively, results in distinct electrical signatures (Caravaca et al. 2017a; Steinberg et al. 2016b; Zanos et al. 2018; Masi et al. 2019). Additionally, motor cortex activity was recorded, decoded, and used to purposefully move a paralyzed patient’s own hand by connecting electrodes to muscles in the arm (Bouton et al. 2016). These discoveries demonstrate that aspects of the electrical signals that arise in the nervous system can be decoded and understood in a useful way. It is conceivable that this knowledge could potentially be used to selectively activate the sensory arc of a neural reflex in order to evoke a specific anti-inflammatory response. We are still only in the beginning of deconvoluting these signals in peripheral nerves, and although the potential applications are very exciting, it will still likely take considerable time before this technology can be standardized and useful outside experimental neuroscience and immunology.

A limitation to studying cardiovascular disease with the use of bioelectronic devices is the lack of chronic, functional electrodes, specifically designed for mouse peripheral nerves. The field is expanding and different strategies are currently being explored to overcome this challenge, implementing innovations from advances in biomaterials and biophysics. In particular, flexible microelectrodes are revealing themselves promising to be used as chronic implants, in both stimulation and recording of nerve activity (Caravaca et al. 2017a). Other methods and approaches are being considered, one of which concerns wireless electrode technology that can be activated by external stimuli. These devices can elicit action potentials upon illumination (Jakešová et al. 2019) or exposure to a magnetic field (Lee et al. 2016). Another alternative strategy for non-invasive nerve stimulation is the use of ultrasound. Delivery of pulsed ultrasound to the spleen stimulates components of the cholinergic anti-inflammatory pathway, and reduced kidney tissue destruction and decreased inflammation after ischemia-reperfusion injury as well as reduced TNF levels in acute endotoxemia (Cotero et al. 2019; Okusa et al. 2017). Further research needs to be performed to assess and integrate non-invasive miniaturized stimulation interfaces in mice.

## Conclusions

Atherosclerosis is a chronic inflammatory disease, and as neural reflexes regulate aspects of both innate and adaptive immunity, vagus nerve stimulation is of interest to explore as a potential therapeutic strategy in cardiovascular disease. Pharmacological and electrical vagus nerve stimulation has been shown to ameliorate inflammation in a wide variety of experimental and some clinical studies of inflammatory diseases. However, the role of nerves and the inflammatory reflex in atherosclerosis is poorly understood. Moving forward, technological progress and expanding knowledge of mechanisms of neural control of vascular inflammation will be instrumental for any potential advancement of bioelectronic medicine into treatment of cardiovascular disease.
